# CHAF1A Promotes Preadipocyte Differentiation and Contributes to Macrosomia in Gestational Diabetes Mellitus

**DOI:** 10.1007/s43032-025-01946-z

**Published:** 2025-08-12

**Authors:** Dandan Xia, Xun Xu, Yuhui Zhang, Chenying Zhang, Huiyan Wang

**Affiliations:** 1 Department of Obstetrics and Gynecology, Changzhou Maternal and Child Health Care Hospital, Changzhou Medical Center, Nanjing Medical University, 16# DingXiang Rd, ZhongLou District, Changzhou, 213000 China; 2 Department of Orthopedics, Changzhou Maternal and Child Health Care Hospital, Changzhou Medical Center, Nanjing Medical University, Changzhou, 213000 China; 3 Changzhou Key Laboratory of Maternal and Child Health Medicine, Changzhou, Jiangsu 213000 China

**Keywords:** CHAF1A, Gestational diabetes mellitus, Macrosomia, Preadipocyte differentiation, Proliferation

## Abstract

Gestational diabetes mellitus (GDM) leads to macrosomia primarily due to fat accumulation caused by adipocyte differentiation. This study aims to investigate the role and underlying mechanisms of Chromatin assembly factor 1 subunit A (CHAF1A) in GDM-induced macrosomia. CHAF1A expression was compared between the GDM with macrosomia group (n = 25) and the normal glucose with normal weight group (*n* = 15), and the correlation between CHAF1A and neonatal body composition was examined. CHAF1A was overexpressed and knocked down in human visceral preadipocytes (HPA-v), and the effects on cell proliferation and adipogenic differentiation were measured, then the expressions of adipogenic markers were determined. Transcriptome sequencing was employed to investigate the potential mechanisms. Placental immunohistochemistry showed that the expression of CHAF1A in the GDM with macrosomia group was significantly higher than that in the control group (*P* < 0.05). Correlation analysis showed that CHAF1A expression was positively correlated with neonatal weight, body fat percentage, and fat mass. In functional assays, preadipocytes overexpressing CHAF1A showed enhanced proliferation and adipogenic differentiation, while knockdown of CHAF1A resulted in the opposite effect. Moreover, CHAF1A affected the expression of adipogenic markers. Transcriptome sequencing analysis showed that the differentially expressed genes after CHAF1A silencing were enriched in signaling pathways closely related to preadipocyte differentiation and hormone secretion and synthesis, such as JAK-STAT, Wnt and BMP signaling pathways. CHAF1A promotes the proliferation and differentiation of preadipocytes, which may be a direction for exploring fetal fat accumulation leading to macrosomia in GDM.

## Introduction

Gestational diabetes mellitus (GDM) is defined as abnormal glucose metabolism first detected or occurring during pregnancy, which significantly increases the risk of adverse outcomes during the perinatal period and postpartum [1]. The incidence of macrosomia in pregnancies complicated by GDM ranges from 25 to 42% [2]. Macrosomic infants are at increased risk of complications such as shoulder dystocia, postpartum hemorrhage, cesarean delivery, and neonatal asphyxia. Moreover, these infants have a higher likelihood of developing metabolic disorders compared to infants of normal birth weight [3].

Research indicates that adipose tissue accounts for 12% to 14% of fetal birth weight, and 46% of changes in neonatal birth weight are attributed to variations in adipose tissue content [4]. Adipocytes are central cells in regulating energy balance and systemic lipid homeostasis [5]. Adipogenesis, the process by which preadipocytes differentiate into mature adipocytes, is a key mechanism underlying fat accumulation and the development of obesity, playing a crucial role in fetal growth and development [6]. In GDM, the fetus is chronically exposed to a hyperglycemia-induced hyperinsulinemic environment, which promotes protein and fat synthesis while inhibiting lipolysis, leading to excessive fetal growth [7]. The primary distinction between macrosomic infants born to mothers with GDM and normal-weight infants is the increased adipogenesis in the former, which is also a significant factor contributing to the higher risk of perinatal complications and long-term metabolic disorders in macrosomic infants. In the hyperglycemic state of GDM, the placenta secretes a variety of cytokines that enter the fetal circulation via the umbilical vein to induce pancreatic islet cell hyperplasia and hypertrophy in the fetus, leading to increased insulin secretion, or fetal hyperinsulinemia [8]. Hyperinsulinemia drives excessive fetal growth, particularly characterized by excessive accumulation of fat throughout the body, resulting in macrosomia. Therefore, we further explored the associations and underlying mechanisms between placental factors in GDM-related macrosomia and fetal adipogenesis.

Chromatin assembly factor 1 subunit A (CHAF1A) is involved in chromatin assembly, DNA replication, regulation of gene expression, and DNA mismatch repair [9, 10]. As a growth-promoting gene, CHAF1A is highly expressed in various tumors and influences the proliferation, metastasis, and invasiveness of tumor cells [11]. This study aimed to investigate the role of CHAF1A in GDM-induced macrosomia., and to investigate the effects of CHAF1A on the proliferation and differentiation of preadipocytes as well as its underlying mechanisms. We discovered that CHAF1A was highly expressed in the placentas of GDM induced macrosomia and that CHAF1A was positively correlated with neonatal body composition parameters. Subsequent overexpression and knockdown experiments in human visceral preadipocytes (HPA-v) revealed that CHAF1A promotes adipocyte proliferation and differentiation. We also preliminarily explored the potential mechanisms by which CHAF1A regulates adipogenesis through transcriptome sequencing. These findings suggest that CHAF1A may participate in the regulation of neonatal birth weight in GDM, provide new insights into the pathogenesis of GDM-related macrosomia and offer potential targets for future intervention strategies.

## Materials and Methods

### Patients and Clinical Samples

We selected patients who received regular prenatal examination and delivered by cesarean section in Changzhou Maternal and Child Health Hospital in 2023, and collected placental tissues within 15 min post-delivery, including 15 cases of normal blood glucose with normal birth weight and 25 cases of GDM with macrosomia. GDM was diagnosed using a standardized 75 g oral glucose tolerance test (OGTT) at 24–28 weeks of pregnancy according to the criteria of the International Association of Diabetes and Pregnancy Study Group [12]. All GDM patients achieved stable blood glucose control through strict diet and exercise management or insulin therapy. Macrosomia referred to a newborn with a birth weight equal to or exceeding 4000 g. The control group were those with normal OGTT results and a newborn birth weight below 4000 g. Exclusion criteria for mothers were as follows: multiple pregnancies, hypertension, history of diabetes prior to pregnancy, thyroid dysfunction, polycystic ovary syndrome, Cushing’s syndrome, pheochromocytoma, conception through assisted reproductive technology, and other pathological conditions. Exclusion criteria for infants were as follows: preterm birth, fetal growth restriction, fetal congenital anomalies, and other pathological conditions. General information is presented in Table 1.

**Table 1:** Primers and sequences

Gene	Direction	Sequence(5’−3’)
CHAF1A	Forward	GATCGAATTCTCAGGATGCA
	Reverse	CCCGTGTGGAGCCGCTTCCG
PPAR-γ	Forward	TGGGTGAAACTCTGGGAGAT
	Reverse	TGTGTCAACCATGGTCATTTCTTG
SREBP1	Forward	CTGACCGACATCGAAGGTGA
	Reverse	AAGTGCAATCCATGGCTCCG
FASN	Forward	GCAAGCTGAAGGACCTGTCT
	Reverse	AATCTGGGTTGATGCCTCCG
LPL	Forward	CCCTGGTCGAAGCATTGGAA
	Reverse	GCTGGTCCACATCTCCAAGT
C/EBPα	Forward	GCCAAGAAGTCGGTGGACAA
	Reverse	ATTGTCACTGGTCAGCTCCA
sh1	Forward	AATTCACAAGCCCGTCTGCCGTTTAACTCGAGTTAAACGGCAGACGGGCTTGTTTTTTG
	Reverse	GATCCAAAAAACAAGCCCGTCTGCCGTTTAACTCGAGTTAAACGGCAGACGGGCTTGTG
sh2	Forward	AATTCCCACCCGGAATGCAGATATTTCTCGAGAAATATCTGCATTCCGGGTGGTTTTTG
	Reverse	GATCCAAAAACCACCCGGAATGCAGATATTTCTCGAGAAATATCTGCATTCCGGGTGGG
sh3	Forward	AATTCAGAAGAAGAGAAGCGCATTAACTCGAGTTAATGCGCTTCTCTTCTTCTTTTTTG
	Reverse	GATCCAAAAAAGAAGAAGAGAAGCGCATTAACTCGAGTTAATGCGCTTCTCTTCTTCTG

### Neonatal Body Composition Analysis

A highly skilled neonatologist conducted anthropometric assessments on 40 newborns within the first 12 h after birth, focusing on measuring skinfold thickness at the biceps, triceps, subscapular, and suprailiac regions. Each measurement was taken three times and then averaged for accuracy. The infants’ body fat mass (FM) and body fat percentage (F%) were estimated using a modified Weststrate method [13]: SFT4 = the sum of bicipital, tricipital, subscapular, and suprailiacal skinfold thickness; body density (D) = 1.1235–0.0719 × log(SFT4); F% = (585/D-550) × 100%; FM = F% × body weight. The characteristics of the neonates are detailed in Table 2.

**Table 2:** The general clinical data of mothers and infants in the two groups

Group	NN(*n *= 15)	GM(*n *= 25)	*P*-value	
Maternal characteristics				
Age (years)	32.93 ± 2.99	31.08 ± 4.23	0.146	
Gestational weeks (W)	38.83 ± 0.84	38.83 ± 1.01	0.985	
Gravidity	3.07 ± 1.39	2.64 ± 1.08	0.283	
Parity	2.07 ± 0.70	1.80 ± 0.71	0.255	
Weight gain during pregnancy (Kg)	11.97 ± 3.50	16.39 ± 5.07	0.005^**^	
Pre-pregnancy BMI (Kg/m^2^)	24.14 ± 3.04	25.06 ± 5.87	0.521	
Weight at delivery (Kg)	71.30 ± 7.87	81.85 ± 14.25	0.012^*^	
OGTT (FBG, mmol/L)	4.42 ± 0.37	5.22 ± 0.67	0.000^***^	
OGTT (1h, mmol/L)	7.65 ± 1.31	10.17 ± 1.27	0.000^***^	
OGTT (2h, mmol/L)	6.83 ± 0.84	8.75 ± 1.45	0.000^***^	
HbA1c (%)	5.01 ± 0.39	6.10 ± 0.51	0.000^***^	
TG (mmol/L)	5.93 ± 1.40	6.17 ± 0.94	0.561	
TC (mmol/L)	4.04 ± 0.83	5.71 ± 2.38	0.003^**^	
Cystatin C	0.96 ± 0.15	1.18 ± 0.25	0.002^**^	
HDL	1.75 ± 0.33	1.71 ± 0.33	0.769	
LDL	3.59 ± 1.06	3.68 ± 0.62	0.769	
Apo A1	1.96 ± 0.35	2.71 ± 0.90	0.001^**^	
Apo B	1.17 ± 0.31	1.34 ± 0.26	0.067	
Lipoprotein a	197.14 ± 78.90	281.74 ± 118.59	0.010^**^	
Free fatty acid	0.41 ± 0.23	0.57 ± 0.0.33	0.104	
Insulin therapy	0(0%)	4(16%)		
Neonatal characteristics				
Gender of newborn			0.341	
Boy	7	15		
Girl	8	10		
Neonatal weight (kg)	3.34 ± 0.24	4.27 ± 0.31	0.000^***^	
Bicipital (mm)	3.67 ± 0.21	4.04 ± 0.15	0.000^***^	
Tricipital (mm)	5.57 ± 0.14	7.55 ± 0.39	0.000^***^	
Subscapular (mm)	5.45 ± 0.16	7.41 ± 0.24	0.000^***^	
Skinfold thicknesses (mm)	5.42 ± 0.17	7.43 ± 0.28	0.000^***^	
Fat nass (FM)(g)	613.97 ± 56.94	975.86 ± 84.51	0.000^***^	
Fat percentage (F%)	18.07 ± 0.44	22.83 ± 0.46	0.000^***^	
SFT4	20.11 ± 0.52	26.44 ± 0.70	0.000^***^	
Body density (D)	1.03 ± 0.0008	1.02 ± 0.0008	0.000^***^	

*NN *Normal blood glucose with normal body weight; *GM *Gestational diabetes mellitus with macrosomia; *BMI *Body mass index; *HbAlc *glycated hemoglobin; *TG *triglycerides; *TC *triglyceride; *HDL *high-density lipoprotein; *LDL *low-density lipoprotein; *Apo A1* Apolipoprotein A1; *Apo B* Apolipoprotein B. SFT4 equals to the sum of bicipital, tricipital, subscapular, and suprailiacal skinfold thicknesses; body density (D) = 1.1235-0.0719 × log (SFT4); FM = F% × body weight; F% = (585/D-550) × 100%

### Cell Lines and Reagents

The human visceral preadipocytes (HPA-v) was purchased from Pronosai (Wuhan, China) and cultured in RPMI 1640 (Gibco, Thermo Fisher Scientific, Waltham, MA, USA) supplemented with 10% fetal calf serum (Gibco, USA). The cell culture conditions were constant temperature at 37 °C and 5%CO2.

### Immunohistochemistry (IHC)

For IHC, placental tissue sections were prepared and processed at room temperature, then deparaffinized and rehydrated for antigen retrieval. Non-specific binding was blocked with 10% goat serum. Sections were incubated with anti-CHAF1A primary antibody (17,037–1-AP, 1:200, Proteintech) overnight at 4 °C, followed by biotinylated secondary antibody for 1 h at room temperature. Immune complexes were detected using DAB, and sections were counterstained with hematoxylin. Positive expression was assessed by multiplying staining intensity and the proportion of positive cells. Staining intensity is divided into four levels: 0, no positive staining(negative); 1, light yellow (weak positive); 2, brownish-yellow (positive); 3, brownish-brown (strong positive). The proportion of positive cells is divided into four ranges: 0, no staining of cells; 1, 1–25%; 2, 26–50%; 3, 51–75%; and 4, 76–100% of cells stained. A score of ≤ 6 points is considered low expression, while a score of > 6 points is defined as high expression.

### Overexpression and Knockdown of CHAF1A

CHAF1A was overexpressed or knocked down by recombinant lentiviral vectors, which incorporates an open reading frame for Green fluorescent protein (GFP). The overexpression sequence:(https://www.ncbi.nlm.nih.gov/nuccore/NM_005483.3?from=124&to=2994&report=fasta;NCBIReferenceSequence:NM_005483.3). The shRNA sequences are shown in Table 1. Cells were plated in 6-well plates with 3 × 10^5 cells per well. When the virus confluence reached about 40%, the virus solution was added, gently shaken, and incubated. After 6 to 16 h, the viral medium was replaced with normal medium. After 48 h of infection, puromycin (5 µg/mL) was added to culture for two passages, and the infection efficiency of CHAF1A was verified.

### Western Blot Analysis

Tissues and cells were lysed in cold RIPA buffer with protease and phosphatase inhibitors, then centrifuged at 12,000 rpm for 15 min at 4 °C. Supernatants were collected and quantified using the BCA method. Proteins were boiled in 5 × SDS loading buffer at 100 °C for 10 min, resolved on SDS-PAGE gels, and transferred to PVDF membranes. Membranes were blocked with 5% nonfat milk and incubated with primary antibodies. The primary antibodies included: CHAF1A (1:1000), PPAR-γ (16,643–1-AP, 1:1000, Proteintech), SREBP1 (14,088–1-AP, 1:1000, Proteintech), FASN (10,624–2-AP, 1:1000, Proteintech), CEBPα(29,388–1-AP, 1:1000, Proteintech), LPL (28,602–1-AP, 1:1000, Proteintech) and β-Actin (66,009–1-Ig, 1:1000, Proteintech) as a loading control. The membranes were then incubated with HRP-conjugated secondary antibodies (A0208, 1:1000, Beyotime, Shanghai, China) for 1 h at room temperature. Immunoreactive bands were detected using an ECL system (Amersham Biosciences) and visualized with an image reader. Densitometric analysis was performed using ImageJ software.

### Quantitative Reverse Transcription Polymerase Chain Reaction (qRT-PCR)

Total RNA was extracted using TRIzol reagent (Invitrogen) and subsequently reverse transcribed with the PrimeScript® RT reagent kit with gDNA Eraser (Perfect Real Time) (Takara), following the manufacturer’s protocol. GAPDH served as the endogenous control. The relative expression of target genes was calculated using the 2-ΔΔCt method and normalized to GAPDH expression. Details of the primers are provided in Table 1.

### Cell Proliferation Assay

Cell proliferation was evaluated using the CCK-8 assay (CCK8, Beyotime, Shanghai, China). Cells were seeded into 96-well plates at a density of 2000 cells per well. At specific time points, 10 μl of CCK-8 solution was added to each well and incubated for 2 h to facilitate color development. Absorbance was measured at 450 nm.

### Triglyceride (TG) Content Determination

Triglyceride content was detected by triglyceride content determination kit (ADS-W-ZF013, AIDISHENG, Jiangsu, China). Cells were collected into a centrifuge tube, and the supernatant was discarded after centrifugation. About 5 million cells were added to 1 mL of the extract, and the cells were broken by ultrasound (ice bath, power 200 W, ultrasonic 3 s, interval of 10 s, repeated 30 times). After centrifugation at 12000 rpm for 10 min at 4℃, the supernatant was removed, and the reagents were added sequentially according to the instructions for use to measure the absorbance at 510 nm.

$$\mathrm{TG}(\mathrm{\mu g}/10^4\mathrm{cell})=(({\mathrm A}_{\mathrm{measurement}}-{\mathrm A}_{\mathrm{blank}})\times{\mathrm C}_{\mathrm{standard}}\times{\mathrm V}_2)\div(500\times{\mathrm V}_1\div\mathrm V)\div({\mathrm A}_{\mathrm{standard}}-{\mathrm A}_{\mathrm{blank}}))\times\mathrm D.$$

#### Oil Red O Staining

The Oil Red O solution was prepared by diluting the stock with distilled water (3:2 ratio), mixing, and filtering twice. Cells were fixed in 4% paraformaldehyde for 30 min, then stained with Oil Red O for 10–15 min. After differentiation with 60% isopropanol, nuclei were stained with hematoxylin, differentiated, and sections were mounted with glycerin gelatin for microscopy. Lipid droplets appeared orange to red, and nuclei were blue.

#### Transcriptome Sequencing Analysis

In HPA-v cells, CHAF1A was knocked down using sh1. Total RNA was extracted from differentiated cells and sequenced on an Illumina HiSeq/Novaseq/MGI2000 instrument with a 2 × 150 paired-end configuration. The transcripts were converted to fasta format, indexed, and expression levels were estimated using HTSeq (v0.6.1). Differential expression analysis was performed using the DESeq2 Bioconductor package, with Padj set at < = 0.05 to identify differentially expressed genes. GO terms were identified using GOSeq (v1.34.1), and significant differential expression genes were enriched in KEGG pathways using in-house scripts. (http://en.wikipedia.org/wiki/KEGG).

#### Statistical Analysis

Each experiment was performed in triplicate. Demographic data are expressed as mean ± SDs. Statistical analysis was performed using SPSS 26.0, GraphPad Prism 9.5, and ImageJ. For IHC scores, normality was verified using the Shapiro–Wilk test. Non-normal data were analyzed using the Mann–Whitney U test. The Bonferroni correction was applied for multiple comparisons in qPCR and Western blot analyses. Comparisons between two groups were made using Student’s t-test or Mann–Whitney U test, while ANOVA followed by Tukey’s HSD test was used for comparisons among three or more groups. Pearson correlation coefficient analysis was used for correlation analysis. *P* < 0.05 was considered statistically significant.

## Results

### High Expression of CHAF1A in the GDM with Macrosomia Group

**Fig. 1:**
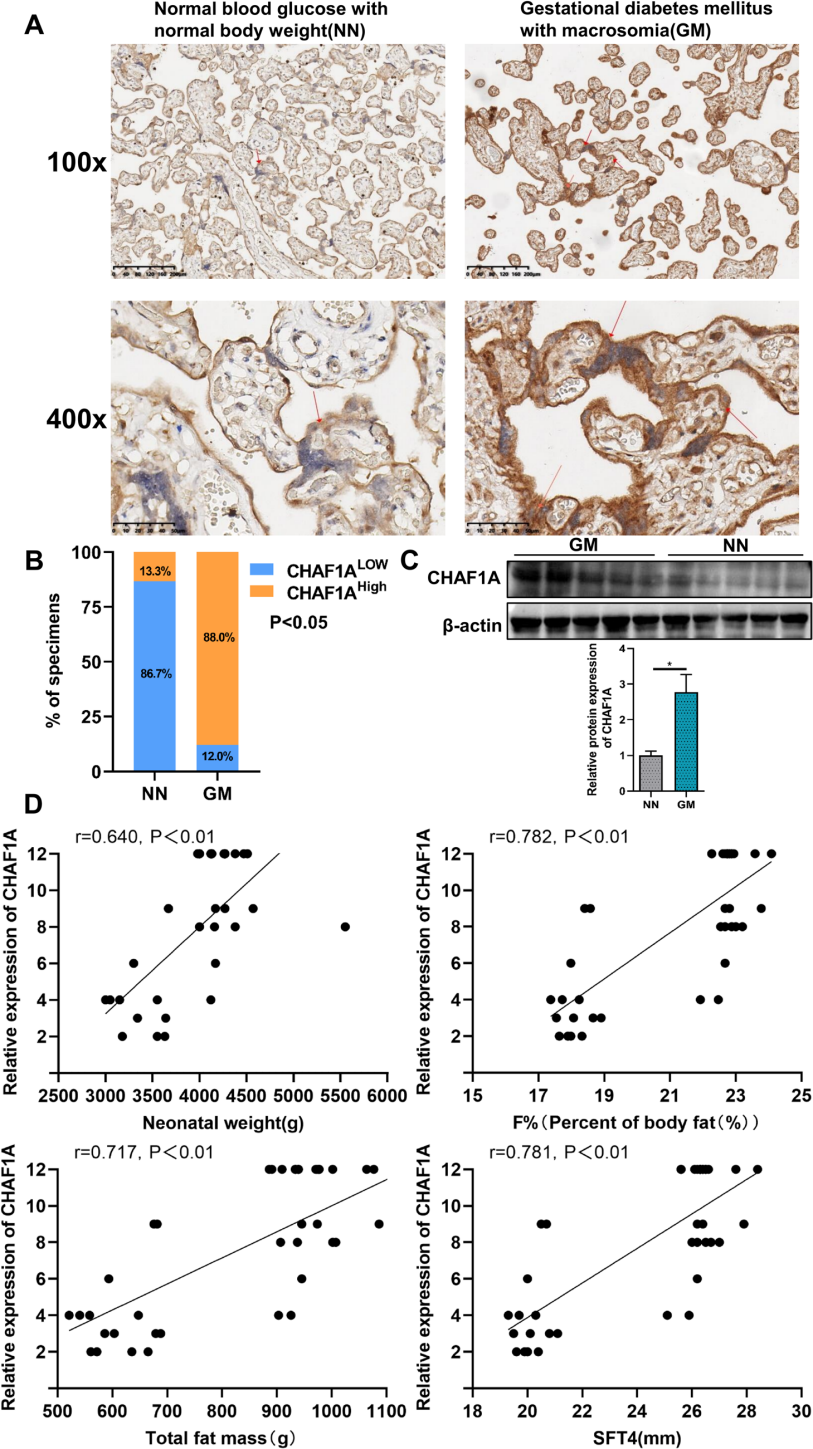
Expression of CHAF1A and its correlation with neonatal body composition. **A** Immunohistochemistry of CHAF1A expression; **B** Analysis of immunohistochemical results; **C** Western blotting of CHAF1A expression; **D** Pearson’s coefficient correlation analysis. *NN* Normal glucose with normal weight group; *GM* Gestational diabetes mellitus with macrosomia; *F% *body fat percentage; *SFT4 *the sum of bicipital, tricipital, subscapular, and suprailiacal skinfold thickness

A total of 15 placentas from the normal glucose with normal weight group and 25 placentas from the GDM with macrosomia group were collected. Immunohistochemistry revealed significantly higher expression of CHAF1A in the GDM with macrosomia group (*P* < 0.05, Fig. 1A-B). Western blot analysis of placental tissue also indicated that the expression of CHAF1A was increased in the GDM with macrosomia group (*P* < 0.05, Fig. 1C).

### Clinical Data of Maternal and Newborns

There were no differences in maternal age, gravidity, parity, gestational age at delivery, and newborn sex between the GDM with macrosomia group and the control group (P > 0.05). Pregnant women in the GDM with macrosomia group had higher gestational weight gain, weight at delivery, OGTT results, glycated hemoglobin, serum triglycerides, cystatin C, apolipoprotein A1, and lipoprotein-a compared to the control group (*P* < 0.05). Pre-pregnancy BMI, total cholesterol, apolipoprotein B, and low-density lipoprotein levels were also higher in the GDM with macrosomia group, but without statistical significance. The insulin usage rate in the GDM with macrosomia group was significantly higher. In terms of newborn body fat content measurement, birth weight, biceps, triceps, subscapular, suprailiac skinfold thickness, SFT4, FM, and F% were all significantly higher in the GDM with macrosomia group (*P* < 0.01) (Table 2).

### Correlation Between CHAF1A Expression and Neonatal Body Composition

Pearson correlation analysis indicated a positive correlation between newborn weight, F%, FM, and SFT4 with CHAF1A expression levels (newborn weight: r = 0.640, *P* < 0.01; F%: r = 0.782, *P* < 0.01; FM: r = 0.717, *P* < 0.01; SFT4: r = 0.781, *P* < 0.01) (Fig. 1D).

### Effects of CHAF1A Overexpression on the Proliferation and Adipogenic Differentiation in Preadipocytes

**Fig. 2:**
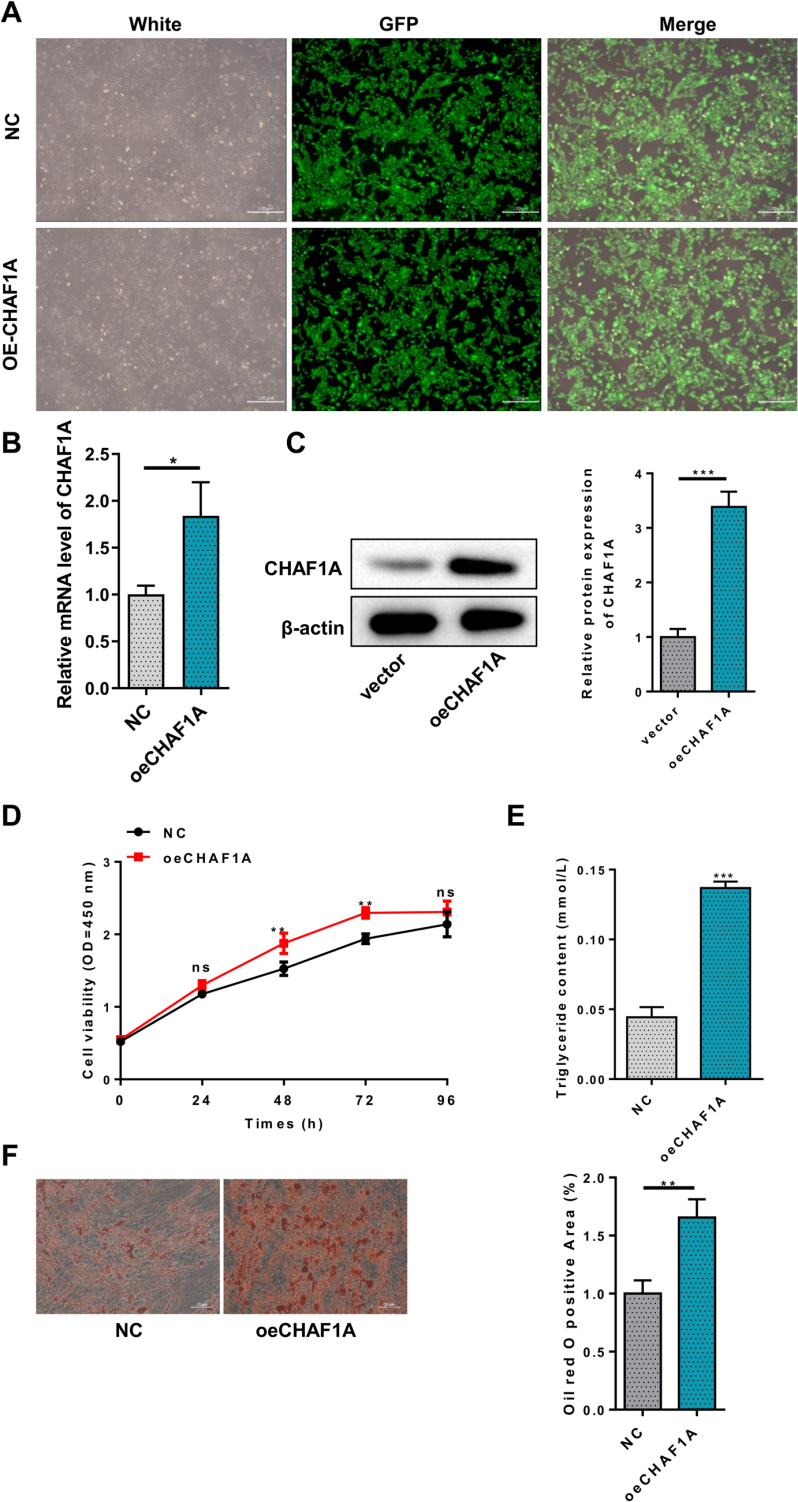
Effects of CHAF1A overexpression on preadipocyte proliferation and differentiation. **A** Cell morphology under fluorescence microscope; **B** CHAF1A mRNA; **C** Western blotting; **D** CCK8 assays; **E** Glycerin triglyceride content assays; **F** Oil Red O staining. ***P < 0.001, **P < 0.01, *P < 0.05

**Fig. 3:**
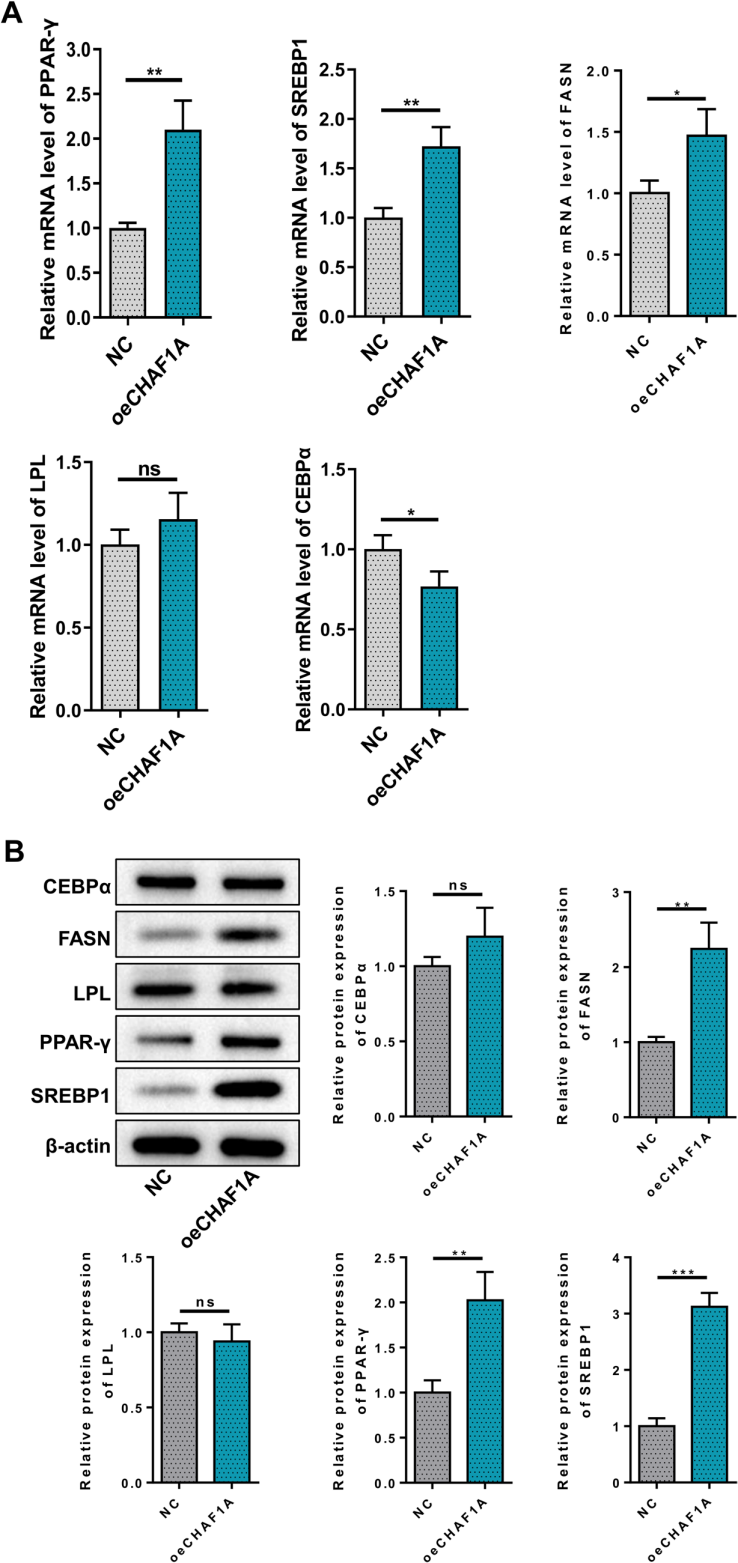
Effects of CHAF1A overexpression on key factors involved in preadipocyte differentiation. **A** PPAR-γ, SREBP1, FASN, CEBPα, and LPL mRNA; **B** Western blotting. ***P < 0.001, **P < 0.01, *P < 0.05

CHAF1A was overexpressed in HPA-v using a lentiviral vector. No significant differences in cell morphology were observed between groups under fluorescence microscopy.(Fig. 2A). CHAF1A was significantly increased at both the mRNA and protein levels after transfection with OE-CHAF1A (Fig. 2B-C). CCK8 assays revealed significantly enhanced cell proliferation at 48 h and 72 h post-transfection (Fig. 2D). Subsequently, preadipocytes stably infected with CHAF1A overexpression lentivirus or negative control lentivirus were induced to differentiate. Glycerin triglyceride content assays indicated significantly higher levels in the OE-CHAF1A group (Fig. 2E). Oil Red O staining showed a marked increase in lipid droplet aggregation after OE-CHAF1A transfection (Fig. 2F). We then assessed the impact of CHAF1A overexpression on several key factors involved in preadipocyte differentiation. qPCR results showed significantly elevated mRNA expression of PPAR-γ, SREBP1, FASN, and CEBPα after OE-CHAF1A transfection, with no significant change in LPL mRNA levels (Fig. 3A). Western blot showed significantly increased protein expression of PPAR-γ, SREBP1, and FASN, with no significant change in LPL and CEBPα protein levels (Fig. 3B).

### Effects of CHAF1A Knockdown on the Proliferation and Adipogenic Differentiation of Human Preadipocytes

**Fig. 4:**
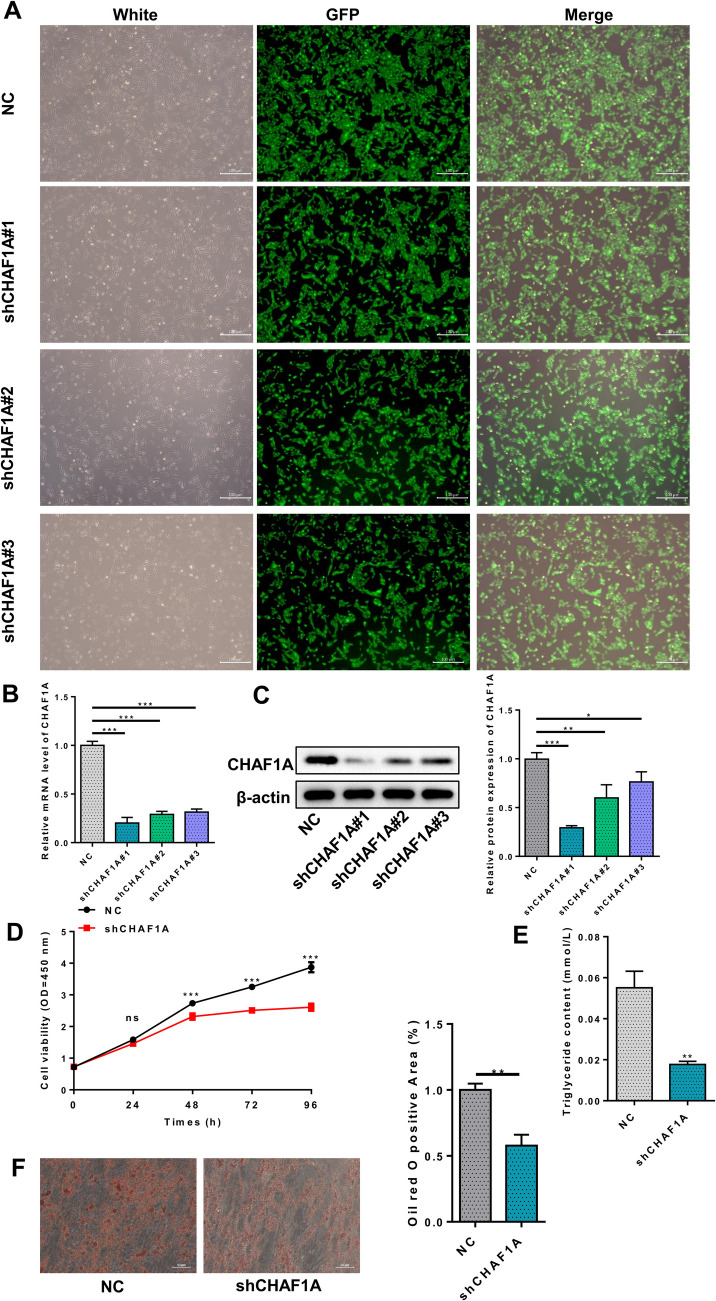
Effects of CHAF1A knockdown on preadipocyte proliferation and differentiation. **A** Cell morphology under fluorescence microscope; **B** CHAF1A mRNA; **C** Western blotting; **D** CCK8 assays; **E** Glycerin triglyceride content assays; **F** Oil Red O staining. ***P < 0.001, **P < 0.01, *P < 0.05

**Fig. 5:**
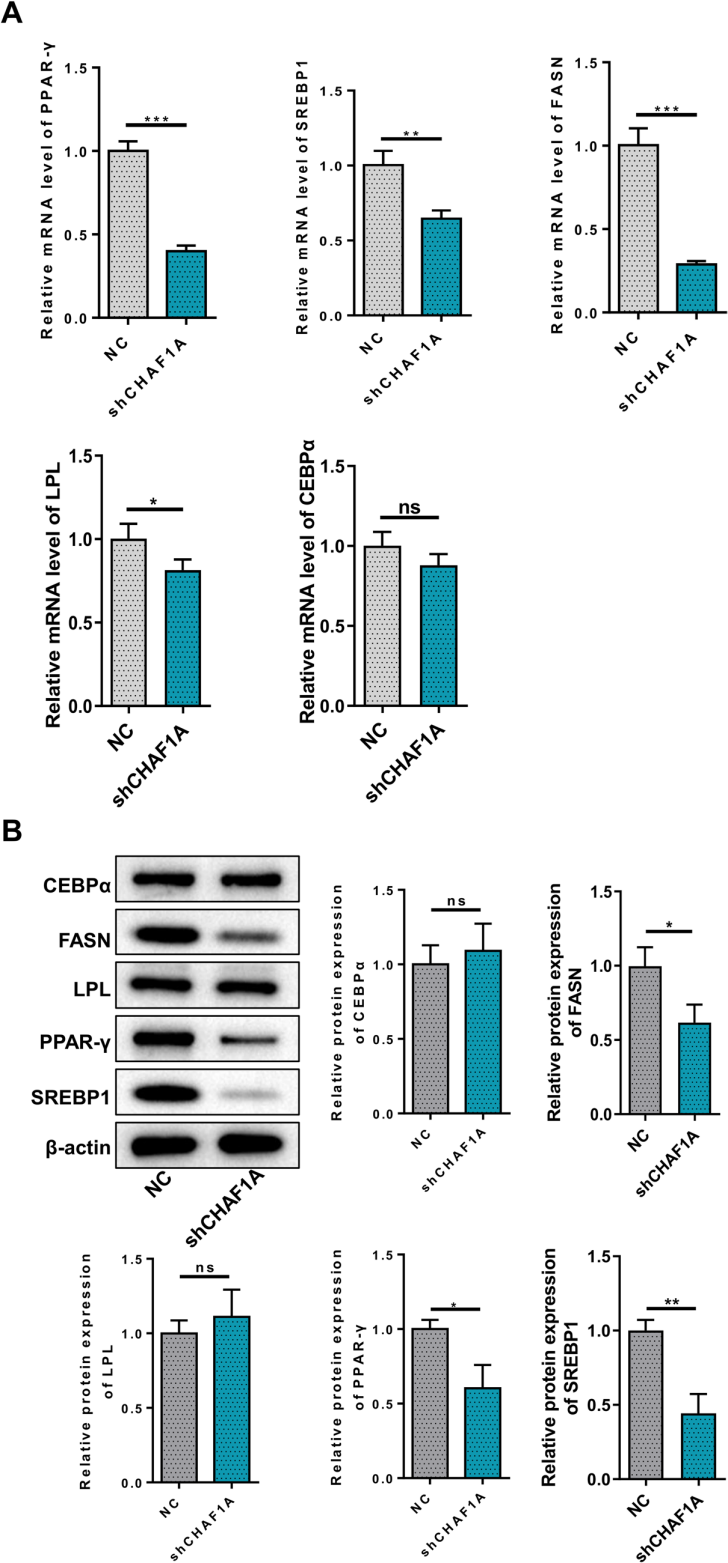
Effects of CHAF1A knockdown on key factors involved in preadipocyte differentiation. **A** PPAR-γ, SREBP1, FASN, CEBPα, and LPL mRNA; **B** Western blotting. ***P < 0.001, **P < 0.01, *P < 0.05

Three shRNAs were used to knock down CHAF1A expression in HPA-v cells (Fig. 4A). Results indicated significant reduction in CHAF1A at both mRNA and protein levels, with sh1 showing the most pronounced effect, thus it was selected for subsequent experiments (Fig. 4B-C). CCK8 assays revealed significantly decreased cell proliferation at 48 h, 72 h, and 96 h post-transfection with sh1 compared to the control group (Fig. 4D). Glycerin triglyceride content assays showed significantly lower levels in the sh1 group (Fig. 4E). Oil Red O staining indicated a marked reduction in lipid droplet aggregation (Fig. 4F). qPCR results showed significantly decreased mRNA expression of PPAR-γ, SREBP1, FASN, and LPL after sh1 transfection, with no significant change in CEBPα gene expression levels (Fig. 5A). The protein expression of PPAR-γ, SREBP1, and FASN after sh1 transfection showed significantly reduced, with no significant change in LPL and CEBPα protein expression levels (Fig. 5B).

### Analysis of Transcriptome Sequencing Results Post CHAF1A Knockdown

**Fig. 6:**
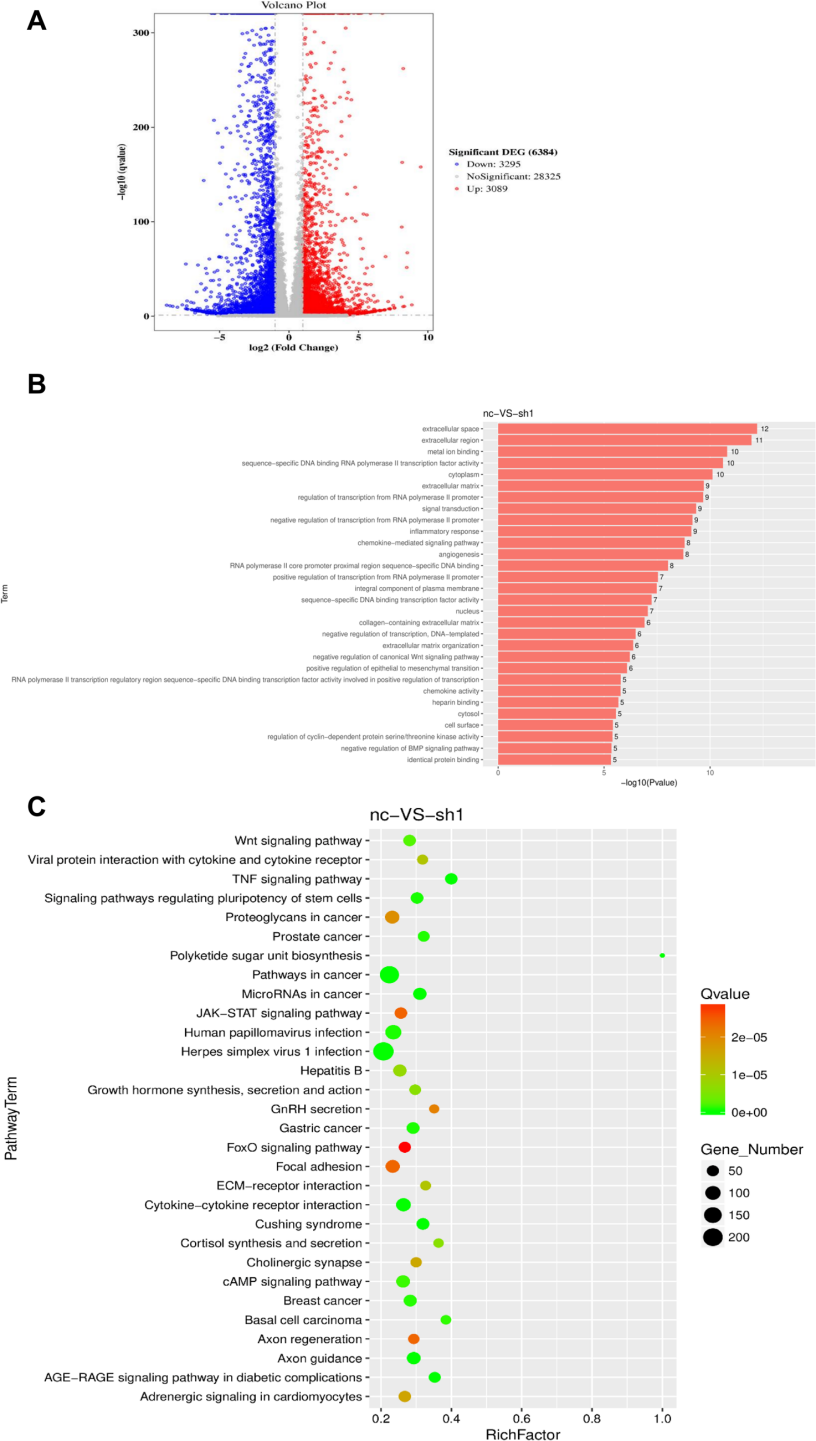

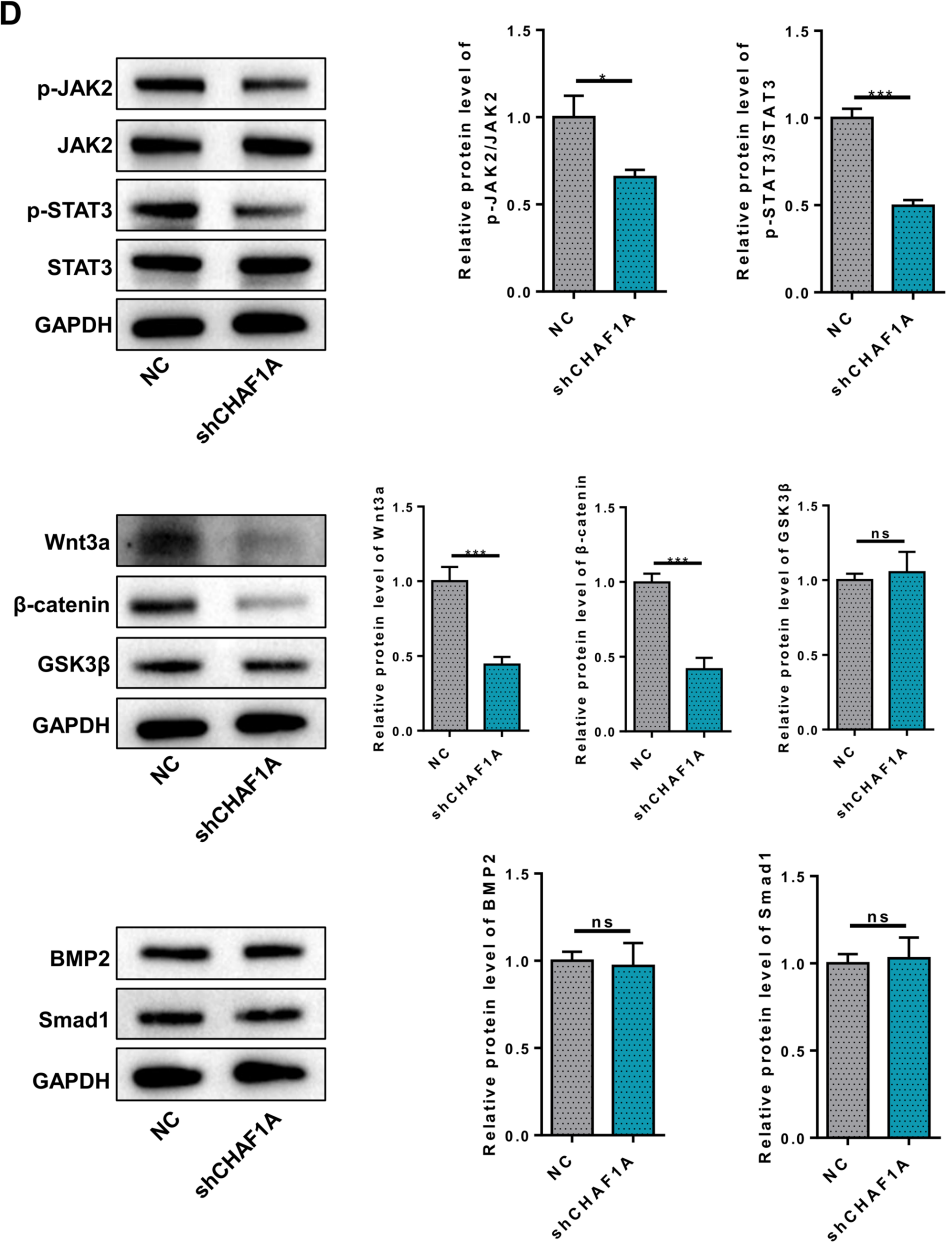
Analysis of transcriptome sequencing results after CHAF1A knockdown. **A** Intersection analysis of differential genes; **B** Annotation of top 30 pathways possibly involved by GO analysis; **C** Annotation of the top 30 pathways possibly involved by KEGG analysis; **D** Western blotting of related pathways. ***P < 0.001, *P < 0.05

Gene chip analysis was conducted on adipocytes with silenced CHAF1A expression to understand the mechanisms by which CHAF1A regulates adipocyte proliferation and differentiation. Transcriptome sequencing analysis revealed that compared to the control group, 3089 genes were upregulated and 3259 genes were downregulated in the sh1 group (Fig. 6A). GO and pathway analysis showed that the differentially expressed genes were enriched in cytokine-cytokine receptor interaction, cAMP Signaling pathway, JAK-STAT signaling pathway, Wnt signaling pathway, signaling pathways regulating pluripotency of stem cells, BMP signaling pathway, etc. (Fig. 6B). These processes have been reported to be closely related to preadipocyte differentiation. KEGG analysis found that differentially expressed genes were also involved in the secretion and synthesis of various hormones, including growth hormone and cortisol, all of which are closely related to growth and development (Fig. 6C). Preliminary Western Blot analysis revealed the JAK2/STAT3 pathway expression, Wnt3a and β-catenin expression decreased following CHAF1A interference. There was no difference in the BMP pathway-related proteins (Fig. 6D).

## Discussion

With the improvement of socioeconomic levels and living standards, the incidence of macrosomia has significantly increased. According to literature reports, 7% to 9% of newborns have a birth weight of ≥ 4000 g [14]. Macrosomia not only increases the risk of dystocia, cesarean delivery, genital tract injury, postpartum hemorrhage, and infection for mothers but also poses short-term complications for neonates, such as birth trauma and asphyxia. Moreover, macrosomia is associated with an increased risk of obesity in childhood and the development of diabetes, and hypertension in adulthood [15].

Maternal lipid and glucose metabolism during pregnancy are crucial factors influencing fetal growth. Gestational diabetes mellitus (GDM), the most common endocrine disorder in pregnancy, is one of the significant contributors to macrosomia [16]. One of the core mechanisms underlying this association may be the excessive accumulation of adipose tissue in the fetus due to GDM. During adipogenesis, the differentiation of preadipocytes into mature adipocytes is a key process for fat accumulation, leading to an increase in both the number and size of adipocytes and contributing to macrosomia and metabolic abnormalities. Key transcription factors such as peroxisome proliferator-activated receptor gamma (PPAR-γ), sterol regulatory element-binding protein 1 (SREBP-1), and fatty acid synthase (FASN) play crucial roles in this process [17]. The chromatin assembly factor 1 subunit A (CHAF1A) is a key gene responsible for bridging cell cycle progression and activation of the DNA damage checkpoint. It can lead to sustained cell proliferation, resistance to cell death, increased genomic instability, and ultimately the formation of a more aggressive cancer phenotype. CHAF1A has been identified as an oncogene in various tumors, such as colorectal cancer and breast cancer, where it is associated with cell proliferation, metastasis, apoptosis, and poor patient prognosis [18].

The placenta serves as an extremely complex endocrine organ, secreting a wide range of bioactive substances, including steroid hormones, neuroendocrine peptides, growth factors, and cytokines. These substances collectively form a highly intricate and sophisticated microenvironment at the maternal–fetal interface [19]. Our study investigated the role of CHAF1A in GDM-induced macrosomia and its regulation of adipogenesis-related factors, revealing that CHAF1A is a key player in adipogenesis and providing new insights into the pathogenesis of GDM-associated macrosomia.

Skinfold thickness is considered an important indicator of increased body fat percentage in neonates born to mothers with GDM. These neonates often exhibit increased total body fat and skinfold thickness at the triceps and scapular areas [20]. GDM-associated macrosomia is typically characterized by metabolic asymmetry, with significant increases in body fat but no marked increase in other tissues/organs. This not only affects infant growth and development but also significantly increases the risk of obesity and diabetes later in life [21]. Our research found that in the GDM-macrosomia group, birth weight, biceps, triceps, scapular, and iliac crest skinfold thickness, subcutaneous fat thickness (SFT4), fat mass (FM), and fat percentage (F%) were all significantly elevated. Moreover, CHAF1A overexpression was positively correlated with neonatal birth weight, F%, FM, and SFT4, suggesting that CHAF1A may play a key role in fat accumulation and weight gain in GDM-induced macrosomia.

Recent studies have shown that in addition to glucose metabolism disorders, GDM patients also experience significant changes in lipid metabolism. For example, plasma levels of free fatty acids (FFA) and triglycerides (TG) are significantly elevated in GDM patients. These metabolic products cross the placenta and enter the fetal circulation, stimulating fetal pancreatic β-cell proliferation and insulin secretion, leading to fetal hyperinsulinemia [22]. Hyperinsulinemia not only inhibits lipolysis but also promotes lipogenesis, further exacerbating fetal adipose tissue accumulation. Additionally, the secretion of various cytokines and hormones in the placenta of GDM patients is altered, influencing fetal metabolic status through the umbilical vein [23]. Studies have found that in GDM patients with well-controlled blood glucose, maternal pre-delivery TG, FFA, and glycerol levels are predictive of neonatal lipid levels and growth [24]. Moreover, postprandial hyperglycemia in GDM can enter the fetal circulation, stimulating fetal pancreatic β-cell proliferation and insulin secretion, leading to fetal hyperinsulinemia. This, in turn, inhibits fetal lipolysis and promotes fat deposition. Increased peripheral tissue fat deposition in the fetus can also lead to insulin resistance, further exacerbating fetal lipid metabolism disorders [25].

PPAR-γ is a nuclear receptor that primarily acts as a transcription factor to regulate adipocyte differentiation and lipogenesis. It is activated by binding to fatty acids and their derivatives, promoting the conversion of preadipocytes to mature adipocytes. In GDM, maternal hyperglycemia and dyslipidemia lead to increased placental transfer of fatty acids, which activate PPAR-γ in fetal cells, promoting adipocyte differentiation and fat accumulation [26]. Adipose tissues produce various protein hormones and inflammatory cytokines including adiponectin and leptin, which positively regulate insulin sensitivity, and resistin, interleukin-6, and tumor necrosis factor-α, which negatively regulate insulin sensitivity. The expression of these adipokines is largely controlled by PPAR-γ [27]. SREBP-1 regulates the expression of lipid synthesis-related genes, including FASN and acetyl-CoA carboxylase(ACC), promoting glycerol triester synthesis within adipocytes [28]. FASN is a key enzyme in fatty acid synthesis, responsible for converting acetyl-CoA and malonyl-CoA into fatty acids, thereby promoting lipid accumulation and adipocyte growth [29]. The coordinated action of these factors leads to increased adipocyte number and size, ultimately increasing the risk of macrosomia. Our study revealed that CHAF1A overexpression significantly enhanced the mRNA and protein levels of PPAR-γ, SREBP-1, and FASN, promoting adipocyte differentiation and triglyceride accumulation. These findings suggest that CHAF1A plays a crucial role in the early stages of adipogenesis and lipid synthesis, which are key processes in fetal fat accumulation and the development of macrosomia in pregnancies complicated by GDM. However, the effects on CCAAT/Enhancer binding protein α (CEBPα)and Lipoprotein Lipase (LPL) were less pronounced. One possible explanation lies in the distinct regulatory mechanisms governing the expression of these genes. PPARγ and FASN are key transcription factors and enzymes, respectively, involved in the early stages of adipogenesis and lipid synthesis. The regulation of these factors by CHAF1A suggests that CHAF1A may play a role in the early commitment of preadipocytes to the adipogenic lineage, thereby contributing to the excessive fat accumulation observed in GDM pregnancies. CEBPα is essential for the terminal differentiation of adipocytes [30], while LPL is involved in the uptake and hydrolysis of circulating triglycerides [31]. The lack of significant changes in CEBPα and LPL protein levels upon CHAF1A overexpression or knockdown suggests that these markers may be regulated by other factors or pathways that are not directly influenced by CHAF1A. Another possible explanation is the presence of compensatory mechanisms that maintain the expression of CEBPα and LPL. These markers may be regulated by redundant pathways that ensure their expression even when CHAF1A is perturbed. In summary, the differential regulation of PPARγ/FASN versus CEBPα/LPL by CHAF1A may reflect the distinct roles and regulatory mechanisms of these markers in adipogenesis. Our findings suggest that CHAF1A promotes the early stages of adipogenesis and lipid synthesis. Future studies should focus on elucidating the specific pathways and interactions that mediate the effects of CHAF1A on adipogenic markers.

To further explore the molecular mechanisms underlying CHAF1A-regulated adipogenesis, we performed transcriptome sequencing to analyze gene expression changes following CHAF1A silencing. The results showed that differentially expressed genes were enriched in several signaling pathways closely related to adipogenesis, including cytokine-cytokine receptor interaction, cAMP signaling pathway, JAK-STAT signaling pathway, Wnt signaling pathway, signaling pathways regulating pluripotency of stem cells, and BMP signaling pathway. These pathways play important roles in adipocyte proliferation, differentiation, and hormone secretion and synthesis. The JAK-STAT pathway is a core signaling pathway for cytokine signaling, capable of transducing signals from cytokines, growth factors, and hormones to the nucleus to regulate gene transcription. After JAK activation, STAT phosphorylation forms dimers that enter the nucleus, bind to the promoters of target genes, and regulate gene transcription. The JAK2/STAT3 pathway can regulate inflammation, insulin resistance, and maintain glucose and lipid metabolism stability [32]. In adipocytes, activation of the JAK-STAT pathway upregulates the expression of key transcription factors such as PPAR-γ, accelerating the conversion of preadipocytes to mature adipocytes [33]. The Wnt signaling pathway plays a crucial role in cell fate determination and tissue development. Its inhibition is often associated with the promotion of adipocyte differentiation [34]. The activation of BMP signaling pathway promotes adipocyte maturation [35]. The preliminary results of Western Blot Analysis showed that there was no difference in the expression of BMP pathway-related proteins after interfering with CHAF1A, while the expression of JAK2/STAT3 pathway decreased, the expression of GSK3β in Wnt/β-catenin remained unchanged, and the expression of Wnt3a and β-catenin decreased. However, since only the interference with CHAF1A in HPA-v cells was verified, and no overexpression of CHAF1A in HPA-v cells was tested, further supplementary experiments are needed in the future, and consideration should be given to adding pathway inhibitors or agonists to further search for the key pathways regulated by CHAF1A.

Moreover, differentially expressed genes also involve hormones closely related to growth and development, such as growth hormone and cortisol. Growth hormone is a peptide hormone that primarily promotes the synthesis of various tissues, especially proteins, and plays a role in growth and development [36]. Cortisol effectively promotes the breakdown of tissues and maintains gluconeogenesis by inhibiting the uptake of glucose by peripheral nerve tissues, ensuring dynamic glucose homeostasis in the body [37]. Increased secretion and synthesis of these hormones may further exacerbate the anabolic metabolism of adipocytes, thereby promoting adipocyte differentiation and lipid accumulation, leading to macrosomia.

Our study has some limitations. Firstly, the small sample size in our immunohistochemical studies limits the statistical power and generalizability of our findings. Future research should aim to increase the sample size, and multi-center studies could be considered to enhance the applicability of the results. Secondly, we only focused on the expression of CHAF1A in the placenta. The process and mechanism by which CHAF1A crosses the placental barrier and exerts its effects on neonatal metabolism need to be further studied. Future studies should also evaluate the association of CHAF1A expression during pregnancy with metabolic alterations in GDM women and the differential expression of CHAF1A in neonates. Meanwhile, although we have identified downstream genes involved in differential expression, the specific genes or mechanisms involved in CHAF1A regulation of adipocyte differentiation remain unclear. Finally, further construction of animal models is needed to confirm our conclusions.

## Conclusions

In conclusion, we found that CHAF1A expression was higher in placentas of GDM women with macrosomia, and positively correlated with neonatal body composition. Overexpression of CHAF1A significantly promotes the proliferation and adipogenic differentiation of preadipocytes. The differentially expressed downstream genes after CHAF1A interference are closely related to the biological processes related to adipocyte differentiation and the secretion and synthesis of hormones related to growth and development. In the future, direct evidence from fetal adipose tissue and transplacental studies will be needed to clarify the specific pathways and mechanisms by which CHAF1A influences fetal fat production, thereby providing new insights into the pathogenesis of GDM-related macrosomia.

## Data Availability

The datasets generated and analysed during the current study are available from the corresponding author on reasonable request.
